# Fluorescence Spectroscopy Approaches for the Development of a Real-Time Organophosphate Detection System Using an Enzymatic Sensor

**DOI:** 10.3390/s150203932

**Published:** 2015-02-09

**Authors:** Paola Carullo, Giovanni Paolo Cetrangolo, Luigi Mandrich, Giuseppe Manco, Ferdinando Febbraio

**Affiliations:** The Institute of Protein Biochemistry (IBP) of the Italian National Research Council (CNR), Via P. Castellino 111, 80131 Naples, Italy; E-Mails: p.carullo@ibp.cnr.it (P.C.); g.cetrangolo@ibp.cnr.it (G.P.C.); l.mandrich@ibp.cnr.it (L.M.)

**Keywords:** biosensor, organophosphates, fluorescence spectroscopy, thermostable esterase 2, nerve agents, pesticides

## Abstract

Organophosphates are organic substances that contain a phosphoryl or a thiophosphoryl bond. They are mainly used around the world as pesticides, but can also be used as chemical warfare agents. Their detection is normally entrusted to techniques like GC- and LC-MS that, although sensitive, do not allow their identification on site and in real time. We have approached their identification by exploiting the high-affinity binding of these compounds with the esterase 2 from *Alicyclobacillus acidocaldarius*. Using an *in silico* analysis to evaluate the binding affinities of the enzyme with organophosphate inhibitors, like paraoxon, and other organophosphate compounds, like parathion, chlorpyriphos, and other organophosphate thio-derivatives, we have designed fluorescence spectroscopy experiments to study the quenching of the tryptophan residues after esterase 2 binding with the organophosphate pesticides. The changes in the fluorescence signals permitted an immediate and quantitative identification of these compounds from nano- to picomolar concentrations. A fluorescence based polarity-sensitive probe (ANS) was also employed as a means to understand the extent of the interactions involved, as well as to explore other ways to detect organophosphate pesticides. Finally, we designed a framework for the development of a biosensor that exploits fluorescence technology in combination with a sensitive and very stable bio-receptor.

## 1. Introduction

**Figure 1.**
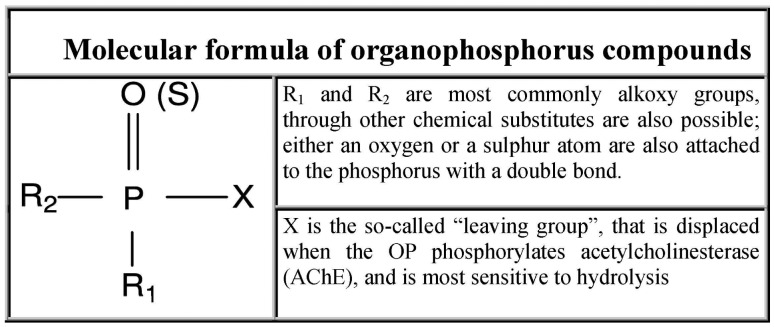
General chemical structure of organophosphate compounds.

Organophosphate (OP) compounds are ester, amide or thiol derivatives of phosphoric, phosphonic or phosphinic acids. Their general formula is shown in Figure 1. These compounds act as irreversible inhibitors of acethylcholinesterase (AChE) that catalyzes the hydrolysis of acethylcholine (ACh), the most important neurotransmitter in the central and peripheral nervous system [1,2], thus affecting the transmission of nerve impulses.

OPs are mainly used around the world as pesticides in agricultural and domestic environments, but some are also chemical warfare agents, the so-called nerve gases or nerve agents, used on the battlefield and in terrorist attacks. In Table 1 the structures of the OPs used in this work, their history and their effects on human health are listed [3–7].

Worldwide pesticide use was estimated at about 5.2 billion pounds in 2007 [8]. Unfortunately, the use of organophosphate pesticides has been steadily increasing in developing countries, particularly in Asia and Latin America, because of the lack of regulation, consumer awareness and effective monitoring programs [9].

Beyond their agricultural use in some African countries, such as Botswana, Mali, Morocco, Ethiopia and South Africa, as well as in countries in the Middle East, including Iran, Iraq, Jordan and Kuwait, it has been estimated that there are more than one thousand tonnes of obsolete pesticide stocks [10], among which OP compounds represent the most significant proportion [11].

Despite the great benefits in terms of protecting crops from pests, in order to feed a growing human population, and also millions of people from malaria and other insect-borne diseases, the widespread use of OP pesticides over the last few years has created serious problems for human health and the environment. In fact, in addition to a significant number of occupational deaths and diseases, especially in developing countries [12], recent studies have indicated several harmful mechanisms of action, even at low OP doses, including interference with neural cell development [13], disruption of nuclear transcription factors [14] and an altered synaptic formation [15], such mechanisms causing a wide range of childhood cognitive and behavioral outcomes, such as lower childhood IQ [16–19] and attention-deficit and hyperactivity-disorder (ADHD)-like behaviors [20,21], including autism spectrum disorders (ASDs) [21,22] (Table 1). For these reasons, the importance of detecting the presence of these compounds in the environment has become more and more clear.

Additionally, it should not be forgotten that OPs are also potential chemical warfare agents. The possibility of terrorist attacks that employ these agents, such as the attack on the Toyko subway with sarin on 20 March 1995 that killed 13 people and injured more than five thousand, provides a further strong reason to continue research on the development of the most effective system for the detection of OPs.

The defense against such agents requires a rapid, sensitive and specific detection in order to limit the affected area and quickly choose the best treatment for intoxicated people, thereby saving lives and preventing serious permanent damage. This cannot be achieved using classic methods for OP detection that use gas chromatography (GC) or liquid chromatography (LC) in association with mass spectrometry (MS) [23–26], as such techniques are too expensive, time consuming, and often not suitable for *in situ* and real time detection.

For these reasons, in the last few decades researchers have directed their efforts toward the development of biosensors for easy and rapid OP detection. Biosensors are self-contained integrated devices that provide specific quantitative analytical information using a “biological recognition element” spatially linked with a transducer element able to convert the (bio)chemical signal, resulting from the interaction of the analyte with the bio-receptor, into an electronic one [27,28].

A large number of biosensors currently developed for OP detection have been designed by exploiting their inhibition effects on AChE activity. Effectively, since 1993 the enzymatic inhibition of AChE has been introduced into the field of biosensing as a tool for the detection of pesticides in the environment and in food, and today these technologies are proving to be potential complements to or replacements for the classic methods of analysis [29]. There are several different types of biosensors based on the AChE inhibition that differ primarily in the type of electrode, immobilization surface and signal transduction technology. With regard to the latter the most widely used techniques are based on electrochemical, optical, potentiometric or amperometric systems. Recent papers have described a very sensitive AChE activity-based biosensor for OP detection. In the Li *et al.* paper, the authors, using a photoelectrochemical biosensor, obtained detection limits (LOD) of 10^−14^ M and 10^−12^ M for paraoxon and dichlorvos, respectively [30]. Mishra *et al.* described in their 2012 paper a novel automated flow-based biosensor for OP determination in milk with LOD of 5 × 10^−12^ M, 5 × 10^−9^ M and 5 × 10^−10^ M for chlorpyriphos, paraoxon and malaoxon, respectively [31]. Although these are very interesting results, this type of system, like most acetylcholinesterase-based biosensors, even those made by exploiting advanced technologies, requires the presence of an acetylcholine-like substrate to measure the variation of AChE residual activity after irreversible OP inhibition. This aspect, in addition to the intrinsic low-stability over time of AChE, makes this type of biosensor not suitable for use in real-time or continuous biosensing in the field, like traditional systems of analysis such as LC- and GC-MS.

In order to develop a system for the continuous biosensing and real-time detection of OPs, we have focused our attention on two principal aspects. The first concerns the technique used, that must allow the continuous measurement of the residual activity of the enzyme, exploiting its intrinsic behaviors and so avoiding the addition of substrates and/or other chemicals. Methodologies of fluorescence spectroscopy can be well adapted to this type of measurement.

However, the fluorescence applications described for the recognition of OPs using an enzymatic system are still linked to the use of an enzyme substrate (AChE), or involve indirect measurements, using probes, of the products of the OP hydrolysis by organophosphorus hydrolase (OPH, Table 2). In this last example, the efficiency of the detection system is greatly reduced due to the slow response and low sensitivity.

Other fluorescence-based applications that exploit chemical substances, like transition metal complexes, or changes in the fluorescence intensity of the indole group after oxidation to an indoxy species, lack specificity and sensitivity [32].

Fluorescence spectroscopy is extremely sensitive, allowing the detection of single molecules in solution [33]. It is an absolutely non-invasive technique that allows the monitoring of the fluorescence emission of appropriate fluorophores inside an organism by measuring signals from the outside of the tissue [34,35] and has been successfully used for *in vivo* sensing [34]. By using fluorescent probes, like 8-anilino-1-naphthalenesulfonic acid (ANS), sensitive to the micro-environmental changes of molecules of biological interest, it has been possible to record conformational variations of biological macromolecules as well as to study their binding or interaction with other analytes by measuring the displacement of the dyes [36,37].

The dependence of the emission properties of ANS on the environment derives from an increase in its permanent dipole moment as a result of the excitation and subsequent relaxation of the environmental dipoles. This leads to a red shift of the fluorescence emission maximum and a decrease in fluorescence intensity in polar media [37].

In this work, we have tested two different fluorescence approaches, exploiting the aromatic amino acid residues (tryptophan, tyrosine and phenylalanine) in proteins which may contribute to the intrinsic fluorescence, as well as an external fluorescence probe, ANS, that is commonly used to investigate molecular assemblies and protein binding interactions.

The second, but no less important, aspect concerns the biocatalytic part of the biosensor to be used in the ongoing monitoring which must show a high lifetime and stability in addition to a high sensitivity and responsiveness. We have already described the possibility of using the esterase 2 from *Alicyclobacillus acidocaldarius* (EST2), as the biological part of a colorimetric biosensor for paraoxon detection, demonstrating its characteristics of stability over time, reproducibility and sensitivity [38].

**Figure 2.**
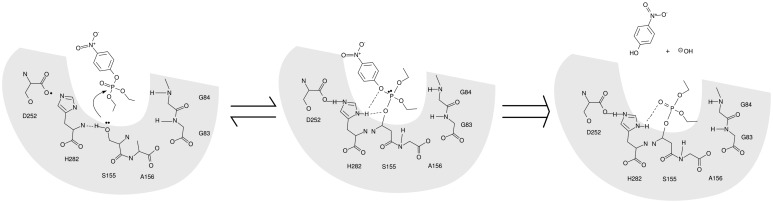
Schematic representation of the EST2-paraoxon reaction.

EST2 is a carboxylesterase belonging to the hormone sensitive lipase (HSL) family that includes several AChEs. This enzyme shows a very long time stability and an appropriate resistance and activity at different pH values and temperatures, [39–41] as well as a good stability in the presence of low concentrations of organic solvents and detergents [42,43]. Furthermore, the EST2 3D structure has been solved at 2.6 Å, [44,45] giving the possibility of having a model structure for *in silico* studies such as molecular docking predictions. EST2 interacts with paraoxon in a similar way to the substrate (Figure 2), but the covalent intermediate of reaction is too stable to dissociate, so irreversibly inhibiting the enzyme.

EST2's peculiarity in terms of stability, in addition to its sensitivity and selectivity toward phosphoryl OPs, like paraoxon and methyl paraoxon [38,46], makes this enzyme a candidate for use as a bio-receptor in biosensors for qualitative and quantitative OP detection. Our final goal is to design a framework for the development of a real-time system for the continuous and on-line detection of OP compounds using fluorescence technology in combination with a high performing bio-receptor.

## 2. Experimental Section

### 2.1. Reagents

All reagents were of analytical grade, and were obtained from commercial sources. 2-[4-(2-Hydroxyethyl)-1-piperazino]-ethansulfonic acid (HEPES), 8-anilino-1-naphthalenesulfonic acid (ANS), diethyl-*p*-nitrophenyl phosphate (paraoxon), diethoxy-(4-nitrophenoxy)-sulfanylidenephosphorane (parathion), diethoxy-(6-methyl-2-propan-2-yl-pyrimidin-4-yl)oxy-sulfanylidenephosphorane (diazinon), 2-(dimethoxyphosphinothioylsulfanylmethyl)isoindole-1,3-dione (phosmet), O,O-diethyl O-3,5,6-trichloro-2-pyridyl phosphorothioate (chlorpyrifos), were all obtained from Sigma-Aldrich (St. Louis, MO, USA).

### 2.2. Enzyme Preparation

EST2 was overexpressed in the mesophilic host *E. coli* strain BL21 (DE3) and purified as previously described in Manco *et al.* [47]. The purity of the enzymatic solution was tested by SDS-PAGE and RP-HPLC. The protein concentration was estimated by the optical absorbance at 280 nm, using a molar extinction coefficient of 1.34 × 10^5^ M^−1^·cm^−1^ in 40 mM sodium phosphate buffer pH 7.1, at 25 °C, as described in Manco *et al.* [48].

### 2.3. Sample Preparation

The stock solutions of the different OPs were prepared by dissolving the compounds in DMSO at the concentration of 10 mM. To reduce the effects of DMSO on the measurements and to avoid in-cuvette precipitations of the compounds, the OP samples were diluted in 50% DMSO at the final concentration of 0.1 mM, and added to the protein sample (0.5 mL) in volumes of 1 mL for a final DMSO concentration of 0.1%.

Aliquots of EST2 at the concentration of 1.2 μM (1.2 nmoles/mL) in 0.5 mL of 40 mM sodium phosphate buffer pH 7.1 were used for the experiments of quenching of the intrinsic fluorescence. Increasing OP concentrations, in the range from 0 to 2 μM, were added to the sample solution, and after 5 s incubation, the emission spectra were acquired.

Fluorescence spectroscopy measurements, by using the ANS fluorescent probe at the final concentration of 50 μM, were carried out on 1.5 μM (1.5 nmoles/mL) EST2 aliquots, in 0.5 mL of 40 mM sodium phosphate buffer pH 7.1. As previously described, an increasing concentration of the different OPs, in the range from 0 to 2 μM, was added to the sample solution, and the emission spectra were acquired after 5 s incubation.

### 2.4. Fluorescence Spectroscopy

Fluorescence spectroscopy measurements were carried out in a FP-8200 spectrofluorimeter (JASCO, Tokyo, Japan) thermostatted by an external Thermo HAAKE K10 (Thermo Fisher Scientific Inc., WALTHAM, MA, USA) thermostatic bath at the temperature of 30 °C, using a quartz cuvette of 1 cm optical path.

The emission spectra of intrinsic fluorescence were recorded in the range from 300 to 450 nm using an excitation wavelength of 280 nm, a 0.5 nm step resolution, and a 100 nm/min scan speed with an accumulation of 3. All the fluorescence measurements were performed in triplicate.

The emission spectra of ANS were acquired in the range from 420 to 600 nm, using the maximum of excitation at the wavelength of 350 nm of the fluorescent probe, with a step resolution of 0.5 nm and a scan speed of 100 nm/min. All the fluorescence spectra were obtained in triplicate. The acquired spectra were imported in the ASCII format, and elaborated using the Qtiplot (Copyright 2004–2009 Ion Vasilief, Craiova, Romania) software.

### 2.5. Docking and 3D Molecular Structure Analysis

Computer simulations were carried out on the EST2 3D crystallographic structure resolved at 2.6 Å (ID number 1EQV from Protein Data Bank [49] (http://www.rcsb.org/pdb/)). The pdb file was opportunely edited to remove the HEPES group that covalently binds to the Ser155 during the crystallization process, from the catalytic site. The docking analysis was carried out using Autodock Vina [50]. In order to determine the grid sizes, visualize the ligand poses and add hydrogen atoms to the template, the ADT software package was employed [51]. The values of the intramolecular distances between the side chains of the aminoacid residues were calculated using the software Swisspdb viewer version 3.7 [52]. The structure images were produced by using the visual molecular dynamics (VMD) [53] software and the Tachyon built-in ray tracing engine.

## 3. Results and Discussion

### 3.1. Fluorescence Spectroscopy and in Silico Analysis of the EST2-OP Complexes

EST2 has shown a very high sensitivity toward inhibition by some OPs such as paraoxon. The measurement of the residual activity of the enzyme after inhibition gives the indirect measurement of the paraoxon concentration [38]. However, it is difficult to use this methodology of detection for real time measurements of OP concentrations in the environment.

In order to transform this detection system into one that can be used in real-time, we envisioned exploiting fluorescence spectroscopy to detect changes in the micro-environment of the aromatic aminoacid residues, in particular the tryptophans that can be directly related to the paraoxon binding to the enzyme.

Fluorescence is a very sensitive technique that permits, by measuring variations in the fluorescence emission intensity compared to a control signal, the development of a fluorescence biosensor able to monitor the interaction between the biomolecule and small ligands present in very low concentrations. When a ligand determines a reduction of the fluorescence signal of a biomolecule it is possible to monitor the increase in the ligand concentration by plotting the ratio between the maximum fluorescence intensity in the absence (F_0_) and presence of a ligand (F).

**Figure 3.**
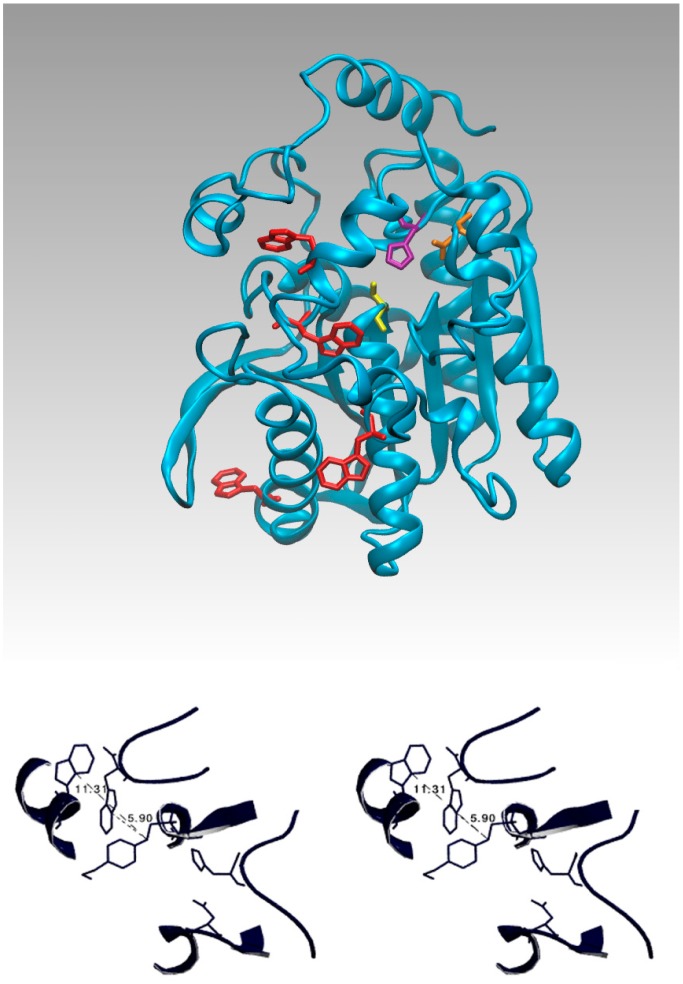
Tryptophan localization in the EST2 3D structure. A schematic representation of the EST2 3D structure, showing in red the tryptophan residues, and in yellow, purple and orange the Ser155, His282 and Asp252 residues, respectively, forming the catalytic triad. Below, a zoomed stereo-view of the EST2 3D structure showing the catalytic triad, the serine bound HEPES inhibitor and the tryptophan residues 85 and 213. The distances are measured from the centroid of the tryptophan eterocycle ring and the oxygen bridging HEPES and Ser155.

**Figure 4.**
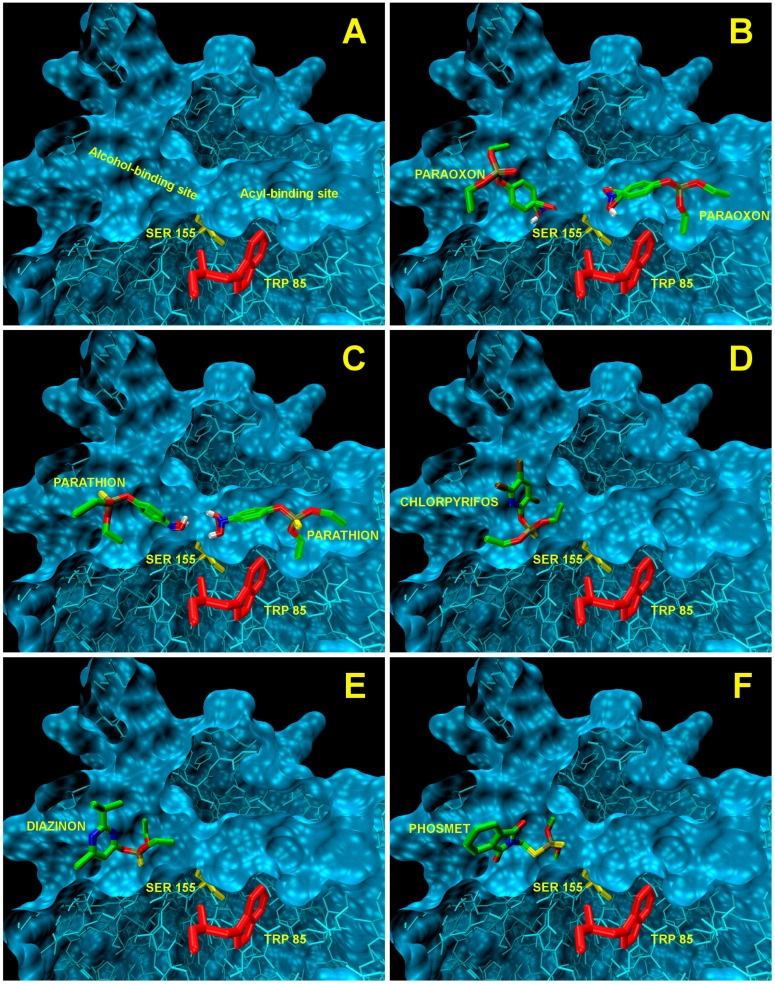
Docking analysis of the EST2-OP interactions. (**A**) Representation of the inside of the EST2 catalytic site. The acyl- and alcohol-binding pockets and the residues of Ser155 (yellow stick) and Trp85 (red stick) are indicated. In the panels from (**B**) to (**F**), a representation of the docking results for the binding of paraoxon, parathion, chlorpyrifos, diazinon and phosmet to EST2, respectively, is shown.

Tryptophans have a stronger fluorescence and a higher quantum yield than the other two aromatic amino acids. In the EST2 sequence only four tryptophan residues are present, internally localized according to the 3D structure (Figure 3A); two of these residues (134 and 229) are located very far from the residues in the active site, while residues 85 and 213 are near the catalytic cavity (Figure 3A). In particular, the indole ring of tryptophan 85 is less than 7 Å from the Ser155-HEPES complex (Figure 3B), being located on the floor at the center of the catalytic tunnel (Figure 4A).

The fluorescence quenching of tryptophan rapidly decreases if the quencher distance increases over 7–8 Å from the centroid of the indole ring of the tryptophan residue, as has been clearly described in literature [54]. Therefore, a contribution of a single tryptophan residue to the intrinsic fluorescence quenching of EST2 after covalent binding with inhibitors appears possible from the structural analysis.

**Figure 5.**
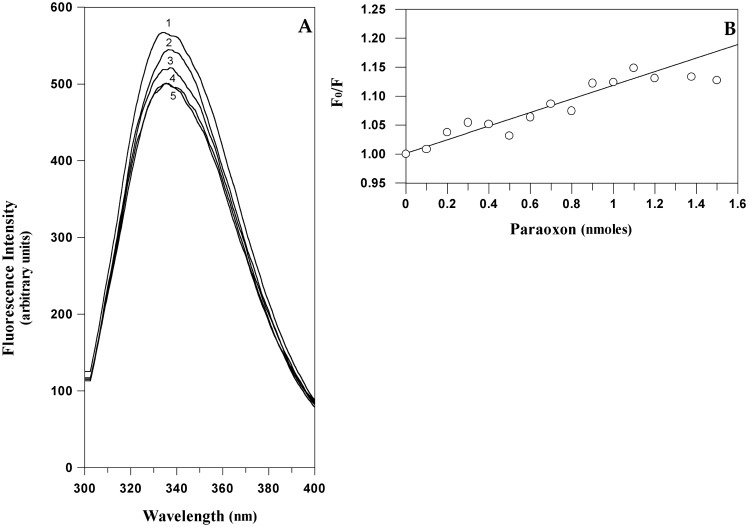
Fluorescence spectra analysis of EST2 in the presence of paraoxon. (**A**) Fluorescence emission spectra of EST2 excited at 280 nm, in the absence (1) and presence of increasing paraoxon concentrations: 0.5 (2); 0.7 (3); 1.0 (4) and 1.2 (5) nmoles; (**B**) Plot of the ratio between the fluorescence intensity, at the maximum of emission in the absence (F_0_) and presence (F) of paraoxon, and the pesticide concentration.

Starting from this hypothesis it could be feasible to quantitatively measure the amount of paraoxon bound to EST2 by a direct measurement of the protein fluorescence. The typical fluorescence emission spectra of EST2 in the 300–450 nm wavelength range, after excitation at 280 nm, showed a maximum at 340 nm (Figure 5A). As expected, the addition of paraoxon to the enzyme solution decreased the fluorescence intensity (associated with the tryptophan quenching) without changes in the wavelength values of the maximum of emission (Figure 5A). These results indicated that the micro-environment of the tryptophans does not change, and therefore we could exclude the possibility of non-specific interactions and associate the fluorescence quenching to a simple steric bulk of the inhibitor in the catalytic site. These data support the structural evidence of a direct effect of the inhibitor on the local fluorescence emission of tryptophan residue 85 neighboring the active site of EST2.

The fluorescence quenching data are reported as plots of F_0_/F *versus* the paraoxon quantity (Figure 5B), where F_0_ and F are the fluorescence intensities in the absence and presence of the quencher, respectively. This is because F_0_/F is expected to be linearly dependent upon the concentration of the quencher [55]. The minimum paraoxon concentration detectable by the measurement of the EST2 fluorescence was 10^−10^ moles, comparable with the results obtained by different techniques [31].

However, the intensity of the fluorescence ratio F_0_/F reaches a plateau at the double of the stoichiometric inhibitor/enzyme ratio, indicating that each enzyme molecule binds exactly two inhibitor molecules. These results are not in agreement with the data obtained from the measurement of the EST2 residual activity [38], indicating a 1:1 stoichiometric ratio inhibitor/enzyme. In order to understand the different results observed from the fluorescence and enzymatic activity measurements, we carried out a computational docking analysis to search for the binding dynamics of the paraoxon molecules on the EST2 structure.

EST2 has two different binding pockets for the acyl and alcohol chains of the ester substrate (Figure 4A). We have already demonstrated that monoacyl esters with a long acyl chain length assume an unfavorable conformation to fit into the acyl-binding site that can only accommodate well alkyl chains with fewer than eight carbon atoms [56]. Thus, esters with a longer alkyl chain accommodate in the alcohol-binding site.

The docking analysis of the site of binding for the paraoxon on the EST2 structure indicated that the inhibitor could be accepted in both binding pockets (Figure 4B). The values of affinity in the kcal/mol obtained from Autodock Vina (Table 3) indicate the higher affinity of paraoxon towards the acyl pocket, resulting in the correct orientation for the covalent bond to the Ser155, in agreement with the irreversible inhibition data [46]. Nevertheless, the affinity value determined for the binding to the other catalytic pocket (alcohol-binding site) indicated a comparable value with respect to the affinity into the acyl-binding site, so suggesting that also the alcohol-binding cavity was filled by a second paraoxon molecule, probably after the covalent bond in the primary site.

These observations are useful to explain the results obtained in the fluorescence measurements, linking the presence of a second paraoxon molecule in the alcohol-binding site to the increase in fluorescence quenching over the EST2-paraoxon stoichiometric ratio 1:1.

These results open up new perspectives in the study of the determination of OP compounds using EST2. The *in silico* evidence that, even if not mediated by a covalent binding, the EST2 catalytic site could accommodate OP compounds led us to use a computational approach in order to explore the possibility of identifying other molecules of the OP family that could interact with EST2.

The analysis carried out using AutoDock Vina on some OP compounds like parathion, phosmet, diazinon and chlorpyriphos indicated the high probability of the accommodation of these molecules in the EST2 catalytic pockets (Figure 4). In particular, similar to paraoxon, also the parathion molecule appears to be accommodated in either catalytic cavity, although the affinity values appeared lower than the paraoxon ones (Table 3). The different affinity values could be explained by the steric hindrance of the sulfur atom which is bigger than the oxygen one.

Chlorpyriphos, diazinon and phosmet appeared instead to be accommodated only in the alcohol-binding site (Figure 4D–F), also with good affinity values (Table 3). The presence of chlorine atoms on the ring of chlorpyriphos, as well as the double ring of the phosmet molecule (Table 1) had a steric impact on the accommodation in the acyl-binding site, while the presence of diazinon only in the alcohol-binding pocket probably could be explained by the presence of opposite carbon atoms of propane and methyl groups on the pyrimidine ring, determining a steric hindrance (Table 1).

**Figure 6.**
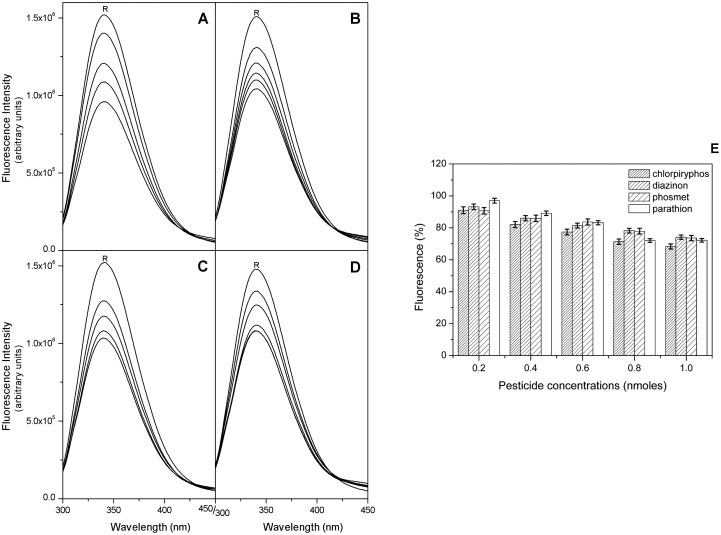
Fluorescence spectra analysis of EST2 in the presence of thiophosphoryl OP. Fluorescence emission spectra of EST2 excited at 280 nm, in the absence (R) and presence of increasing OP concentrations in the range 0.1 to 1.4 nmoles. (**A**) Chlorpyriphos; (**B**) diazinon; (**C**) phosmet and (**D**) parathion; (**E**) EST2 sensitivity towards OPs expressed as % of fluorescence intensity at different concentrations, normalized for the parathion fluorescence.

Starting from the results obtained by the docking analysis, we measured the fluorescence quenching of EST2 in the presence of increasing concentrations of parathion, phosmet, diazinon and chlorpyriphos. The maximum of fluorescence emission decreased after the addition of pesticides for all the analyzed OPs (Figure 6A–D), in agreement with the prediction of their binding to EST2. The plot of the % intensities of fluorescence (Figure 6E) at different OP concentrations, normalized by using the parathion data as a reference, indicated a similar sensitivity toward these compounds, although, even if only slightly, the chlorpyriphos appears to be a better quencher of the EST2 fluorescence.

**Figure 7.**
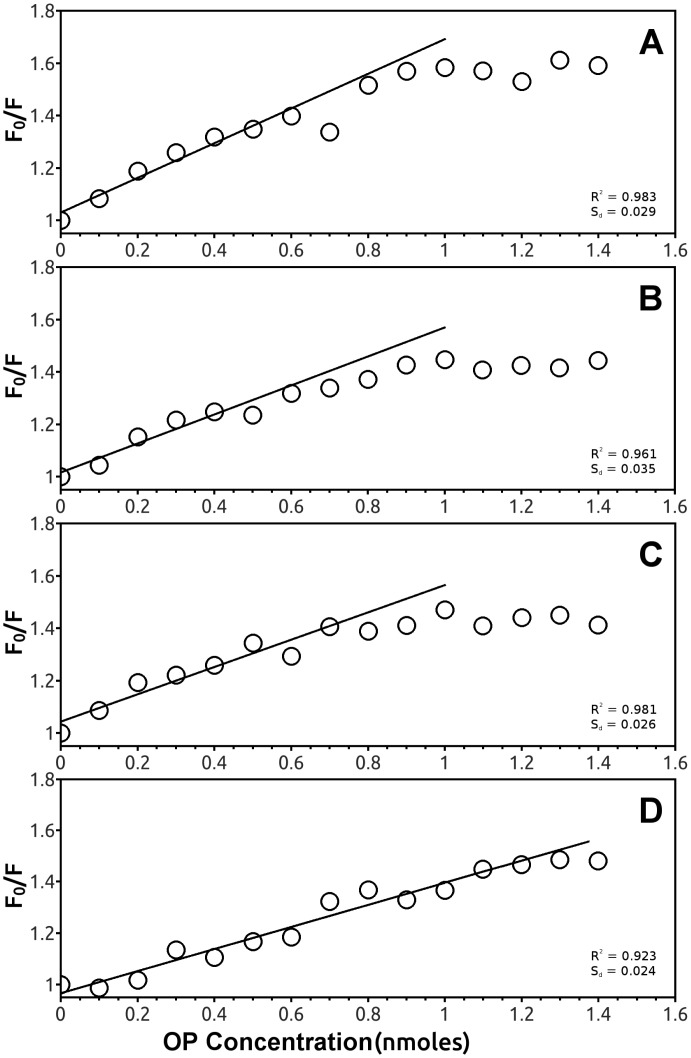
EST2 fluorescence quenching analysis. Plot of the ratio between the fluorescence intensity, at the maximum of emission in the absence (F_0_) and presence (F) of chlorpyriphos (**A**); diazinon (**B**); phosmet (**C**) and parathion (**D**), and the pesticide concentration.

In particular, the F_0_/F plot of EST2 fluorescence in the presence of parathion indicated a 2:1 stoichiometric OPs/enzyme ratio (Figure 7D), while the same plots for the other pesticides reached a plateau at an equimolar stoichiometric ratio (Figure 7A–C).

These results support the evidence that several compounds belonging to the OP family are recognized by EST2, although only a few of them succeed in inhibiting the enzyme thanks to their chemical-physical characteristics, suggesting that the intrinsic fluorescence could be a powerful methodology for pesticide detection.

### 3.2. Fluorescence Spectroscopy Analysis of the EST2-Paraoxon Complex in the Presence of ANS

In order to exploit all the potentiality of this technique, we have considered also the use of fluorescent probes to study the interaction with the OP compounds. In particular, we used paraoxon, that we know covalently binds to the Ser155 in the EST2 catalytic site, as the molecule model in this study.

Considering the presence of several hydrophobic groups covering the walls along the catalytic channel that contains the EST2-paraoxon complex, we employed the extrinsic fluorescent probe ANS. As already described, this probe is sensitive to micro-environmental changes, being essentially non-fluorescent in water, and becoming appreciably fluorescent only when bound to proteins and membranes [37,57,58]. This peculiarity makes it a sensitive indicator of protein folding, conformational changes and other processes that modify the probe exposure to water, like the non-polar cavities in proteins [59,60].

**Figure 8.**
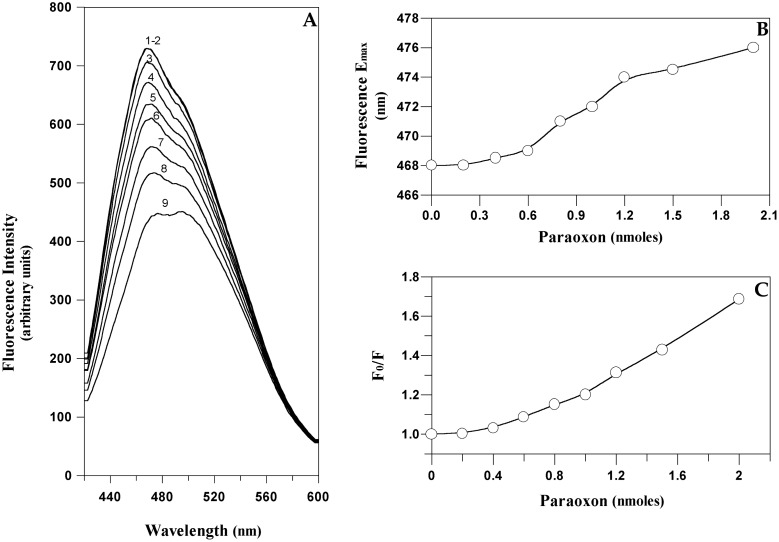
Fluorescence spectra analysis of the EST2-ANS complex. (**A**) Fluorescence emission spectra after excitation at 350 nm of the ANS-EST2 complex in the absence (1) and presence of increasing paraoxon concentrations: 0.2 (2); 0.4 (3); 0.6 (4); 0.8 (5) 1.0 (6) 1.2 (7); 1.5 (8); and 2.0 (9) nmoles; (**B**) Plot of the variation of the maximum emission wavelength at an increased paraoxon concentration; (**C**) Plot of the ratio between the fluorescence intensity, at the maximum of emission of the ANS-EST2 complex in the absence (F_0_) and in presence (F) of paraoxon, and the pesticide concentration.

Fluorescence emission spectra of the ANS-EST2 complex in the range 420–550 nm, excited at 350 nm, showed a maximum at 468 nm (curve 1 in Figure 8A). The addition of paraoxon aliquots to the complex decreased the fluorescence intensity (Figure 8A) and produced a red shift in the maximum of emission (Figure 8B). The observed modifications in the ANS emission spectra did not express the expected variations if only the active site cavity was involved. In particular, when plotting the F_0_/F ratio of the fluorescence intensity at the maximum emission of the ANS-EST2 complex *versus* the different paraoxon concentrations (Figure 8C), the data plot does not reach a plateau at the stoichiometric ratio 2:1 inhibitor/enzyme. This result suggests that the addition of paraoxon and/or the release of the phenol alcohol could cause an increase of the environment polarity, so modifying the fluorescence spectra of ANS in solution, and making impossible a direct determination of paraoxon by this approach. However, the red shift observed in the wavelength of maximum emission suggests different kinds of interaction. In the range of concentrations from 0 to 0.6 nmoles of paraoxon (1:1 stoichiometric ratio inhibitor/enzyme), we observed a slight shift of the value of maximum emission, that increases until 1.2 nmoles of paraoxon (2:1 stoichiometric ratio inhibitor/enzyme), reaching a plateau for higher paraoxon concentrations (Figure 8B).

These data suggest that ANS reacts differently with the binding pockets of the enzyme, which have different non-polar exposed surfaces. Therefore, when the inhibitor covalently binds to the enzyme in the acyl-binding site, the local environmental changes could be outside the catalytic pocket and so have less impact on the overall fluorescence. Conversely, by increasing the paraoxon concentration, the interaction involved the alcohol-binding site, giving a marked local environmental change that affects the ANS properties.

Measuring the red-shift in the ANS fluorescence after the displacement of the dyes by the paraoxon molecules, competing for the binding sites, it became possible to monitor the presence of paraoxon in solution. The advantages in the use of a visible wavelength in terms of ease of handling and the reduced price of the instruments, encourages studies on fluorescent probes, preferably covalently linked to the bio-receptor, for the OP detection using EST2.

## 4. Conclusions

The development of biosensors for OP detection is mandatory, because of their extended presence in the environment as pesticides, and due to the risk of their use as chemical warfare agents. The advantage in exploiting the high selectivity and sensitivity to OPs of some biological macromolecules, compared to chemical methods, directs research toward more robust and stable enzymes, which are resistant/tolerant to most chemicals present in aqueous solutions, and can be used in a large range of temperatures.

The obtained data reinforce the idea that EST2 is an excellent candidate for the development of real-time biosensors able to identify unknown quantities of OPs, because of its high stability, high affinity, selectivity and immediate response time. The use of fluorescence spectroscopy techniques makes it possible to set up a direct measuring system without the use of specific substrates or coupled reactions, with the advantage of a real-time and continuous OP detection.

By integrating a portable spectrofluorimeter with the necessary hardware to support the online measurements of the chip activated with the immobilized EST2, we could obtain a robust sensor with a real world capacity. EST2 is immobilized rapidly and with a high efficiency on nitrocellulose supports [38], and therefore it should be easy to develop protocols for its immobilization on more useful matrices.

The system described could have applications in a number of human activities related to health, such as the continuous monitoring of water contaminants in aqueducts or in waste water, and to safety, such as the continuous monitoring of nerve agents usable for terrorist attacks on significant targets like metros, airports or railway stations.

## Figures and Tables

**Table 1.** Formulas, history and health effects of organophosphate pesticides.

**Name and Molecular Formula**	**History**	**Health Effects**
**Paraoxon** 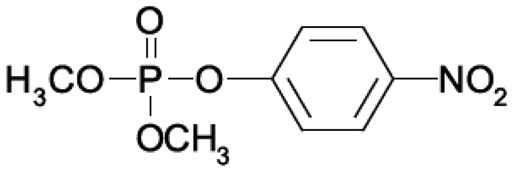	Parathion was developed by Gerhard Schrader for the German trust IG Farben in the 1940s. Because of its high toxicity and risks of exposure to agricultural workers and to birds, and in response to the manufacturers' request, EPA in January 1992 announced the cancellation of all uses of parathion on fruit, nut and vegetable crops. Further, to reduce exposure of agricultural workers, parathion may be applied to these crops only by commercially certified aerial applicators and treated crops may not be harvested by hand. EPA intends to cancel all uses of parathion in the near future	Parathion is readily absorbed into the bloodstream from the skin, lungs or gut. Breathing parathion dusts, or aerosols, may be extremely dangerous. Parathion is rapidly distributed through the body. The liver metabolizes parathion into the active metabolite: paraoxon. It is paraoxon that actually inhibits the cholinesterase. Paraoxon exposure can result in headaches, convulsions, poor vision, vomiting, abdominal pain, severe diarrhea, unconsciousness, tremor, dyspnea, and finally lung-edema as well as respiratory arrest. Symptoms of poisoning are known to last for extended periods of time, sometimes months. Once in the bloodstream, parathion may cross the placenta and is toxic to the fetus and it is a possible carcinogen [3].
**Parathion** 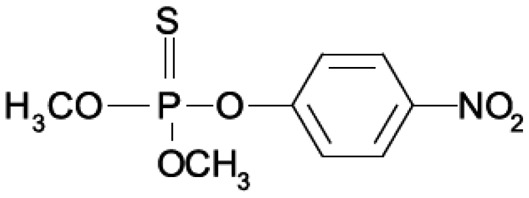		
**Diazinon** 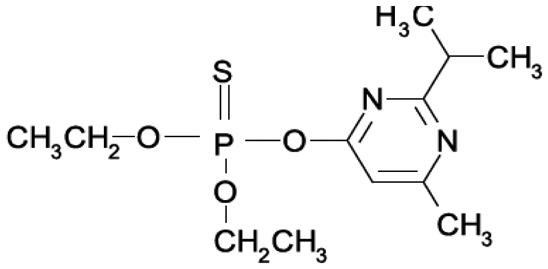	Diazinon was developed in 1952 by the Swiss company Ciba-Geigy as a replacement for the insecticide DDT and it is became an all-purpose commercial pest control product. It is used in flea collars for domestic pets in Australia and New Zealand and approved for use in sheep dip in the United Kingdom. Residential uses of diazinon were outlawed in the USA in 2004 but it is still approved for agricultural uses.	The activation of diazinon is located in the liver. The symptoms on humans are nausea, dizziness, salivation, headache, and rhinorrhea. The symptoms can progress to vomiting, abdominal cramps, diarrhea, muscle twitching, weakness, tremor, a lack of coordination and psychiatric side effects as well as including memory loss and depression. Because of its fat solubility, there is potential for delayed toxicity if significant amounts are stored in fatty tissues [4].
**Chlorpiriphos** 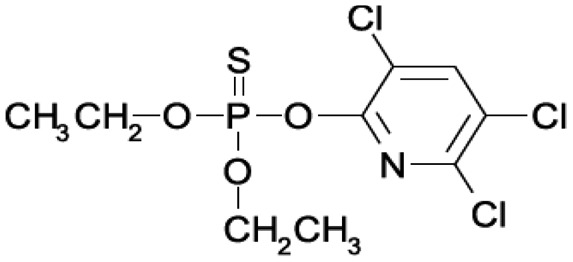	It was introduced in 1965 by Dow Chemical Company. It is registered for use in nearly 100 countries and is applied to 8.5 million crop acres each year. The EPA estimated that between 1987 and 1998 about 21 million pounds of chlorpyrifos were used in the US each year. In 2007, it was the most commonly used in the U. S., with an estimated 8 to 11 million pounds applied [5].	Chlorpyrifos is known by many trade names, including Dursban and Lorsban.. It is moderately toxic to humans, and exposure has been linked to neurological effects, persistent developmental disorders, and autoimmune disorders. Exposure during pregnancy retards the mental development of children, and most use in homes has been banned since 2001 in the USA [6].
**Phosmet** 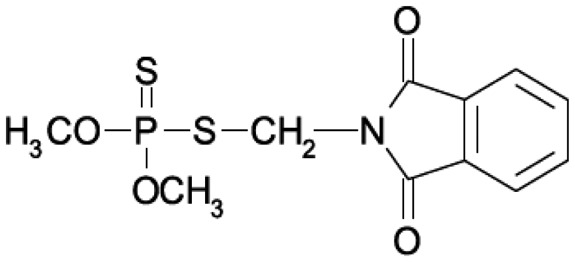	Phosmet was first registered in the USA in 1966 as a broad-spectrum insecticide for control of a wide variety of pests. It is used for direct animal treatments to control fleas, lice, and ticks on cattle, swine and dogs. Since there are many benefits in using the Phosmet, EPA has granted its use for several crops, altought this crop should be located away from residential home and with health protective entry restrictions. It is also necessary a continuous monitoring of the area.	Phosmet is a mild irritant to the eyes and the skin of rabbits. Signs of acute poisoning generally occurring within 30 minutes after exposure. The primary target organ for phosmet is the nervous system. Phosmet is rapidly absorbed, distributed, and eliminated in mammals. Rat studies indicate that phosmet crosses the placenta [7].

**Table 2.** Fluorescence applications for OP detection.

**Bio-Receptor**	**Detection Mechanism**	**Limitations**	**Sensitivity**
Acetylcholinesterase (AChE)	pH-sensitive fluorescent dye detecting the change in pH after substrate hydrolysis.	Indirect measurement	nM
		False positives	
		Slow response time (minutes)	
		Short lifetime due to enzyme degradation.	

Organophosphorus hydrolase (OPH)	pH-sensitive fluorescent dye detecting the change in pH after OP hydrolysis.	Indirect measurement	μM
		Slow response time	
		False positives	
		Short lifetime due to enzyme degradation.	

Monoclonal Antibody	Fluorescence Polarization Immunoassay after formation of antigen-antibody complex.	Indirect measurement	mM
		Very low sensibility	
		Requires baseline testing	

Organophosphorus hydrolase (OPH)	Changes in FRET of the Coumarin 1 after OP hydrolysis.	Indirect measurement	μM

**Table 3.** Values of the best affinity binding of the OPs to EST2 by docking analysis.

**Compound Name**	**Affinity (kcal/mol)**	**Pocket Binding**
Paraoxon	−6.9	acyl
“	−6.4	alcohol
Parathion	−6.0	acyl
“	−5.9	alcohol
Phosmet	−7.0	alcohol
Chlorpyriphos	−5.9	alcohol
Diazinon	−6.2	alcohol
